# Comparative Evaluation of Tissue Attenuation Imaging (TAI) and Ultrasound Attenuation Parameter (UAP) for Noninvasive Quantification and Grading of Hepatic Steatosis

**DOI:** 10.7759/cureus.85117

**Published:** 2025-05-31

**Authors:** Harkaran Singh Mann, Purnoor Mann, Anhad S Mann

**Affiliations:** 1 Radiology, Mann Scanning Centre, Jalandhar, IND; 2 Internal Medicine, Max Institute of Medical Education, New Delhi, IND

**Keywords:** elastography, hepatic steatosis, liver fat quantification, tissue attenuation imaging, ultrasound, ultrasound attenuation parameter

## Abstract

Background and aim: Hepatic steatosis is a major component of chronic liver disease and a key predictor of disease progression. The ultrasound attenuation parameter (UAP) is widely used via transient elastography (TE) for quantifying hepatic fat, but limited access and cost restrict its utility in routine practice. This study aimed to evaluate the correlation between tissue attenuation imaging (TAI) and UAP and to propose reference ranges for grading hepatic steatosis (S0-S3) using TAI as a noninvasive alternative.

Methods: This prospective observational study was conducted at Mann Scanning Centre, Jalandhar, Punjab, India. A total of 120 adult patients undergoing liver evaluation were included. All subjects underwent TE with UAP measurement and ultrasound-based TAI. Steatosis grading (S0-S3) was assigned based on UAP thresholds. Correlation between TAI and UAP was assessed using Spearman's and Pearson's coefficients. Receiver operating characteristic (ROC) curve analysis was performed to derive optimal TAI cutoffs corresponding to each steatosis grade.

Results: TAI showed a strong positive correlation with UAP (Spearman's ρ = 0.61, p < 0.001). TAI values increased progressively across steatosis grades S0 to S3. ROC analysis demonstrated an area under the curve (AUC) of 0.84 for detecting moderate-to-severe steatosis (≥ S2) using TAI. Proposed TAI thresholds for steatosis grading were: S0 (< 0.70 dB/cm/MHz), S1 (0.70-0.79 dB/cm/MHz), S2 (0.80-0.89 dB/cm/MHz), and S3 (≥ 0.90 dB/cm/MHz). The agreement between TAI-based and UAP-based grading was substantial (κ = 0.78).

Conclusions: Tissue attenuation imaging is a reliable and accessible ultrasound-based technique for quantifying hepatic steatosis. It correlates well with UAP and can serve as a practical alternative for steatosis grading in settings where TE is unavailable.

## Introduction

Metabolic dysfunction-associated steatotic liver disease (MASLD), formerly referred to as nonalcoholic fatty liver disease (NAFLD), along with other etiologies of hepatic steatosis, has emerged as a significant global health challenge. The prevalence of hepatic steatosis continues to rise across diverse age groups, paralleling increases in obesity, metabolic syndrome, and diabetes. Hepatic fat accumulation not only plays a pivotal role in the progression of liver fibrosis but also contributes to increased cardiovascular morbidity and mortality and can ultimately lead to advanced complications such as cirrhosis or hepatocellular carcinoma. In this context, the accurate and noninvasive quantification of liver fat has become increasingly important for the diagnosis, risk stratification, and monitoring of affected individuals [1]. The adoption of MASLD terminology is in line with recent international consensus to better reflect the metabolic drivers of the disease [2].

The controlled attenuation parameter (CAP), or ultrasound attenuation parameter (UAP), derived from transient elastography (TE), is an established and validated technique for the quantification of hepatic fat content [3]. However, the implementation of TE requires specialized equipment, which may limit its routine use in many clinical settings, particularly those with constrained resources. Tissue attenuation imaging (TAI), also known as attenuation imaging (ATI), is a newer ultrasound-based technology that enables direct measurement of tissue attenuation using standard ultrasound systems, thereby offering a more accessible and integrated option for clinicians [4,5].

Previous studies have shown that TAI/ATI correlates well with liver fat content when validated against gold standards such as liver biopsy and magnetic resonance imaging (MRI) [6-8]. However, relatively few investigations have focused on defining specific TAI grading thresholds for hepatic steatosis using UAP as the reference standard. The present study was therefore undertaken to evaluate the correlation between TAI and UAP and to establish reference ranges for TAI corresponding to steatosis grades S0 through S3. The broader goal of this research was to promote wider clinical adoption of TAI as a practical and noninvasive tool for liver fat assessment.

## Materials and methods

A prospective observational study was conducted at Mann Scanning Centre, Jalandhar, Punjab, India, between March 2023 and February 2025. During this period, 120 consecutive adult patients who were referred for liver imaging were enrolled. The inclusion criteria specified that participants must be older than 18 years and capable of undergoing both TE with UAP measurement and ultrasound-based TAI. Patients were excluded from the study if they had unreliable UAP measurements, significant ascites, pregnancy, or any chronic illnesses known to affect liver attenuation, such as iron overload.

For each participant, liver stiffness and fat quantification were performed using two modalities: UAP measurements were obtained via the FibroTouch device (Wuxi Hisky Medical Technologies Co. Ltd., Wuxi, China), and TAI was assessed using the Samsung RS85 Prestige ultrasound system (Samsung Medison Co. Ltd., Seoul, Republic of Korea), which is equipped with TAI software. All measurements were performed by experienced radiologists, with UAP and TAI assessments conducted by different operators. To minimize bias, the radiologist performing UAP measurements was blinded to the results of TAI, and vice versa. TAI values, expressed in decibels per centimeter per megahertz (dB/cm/MHz), were obtained from the right lobe of the liver using standardized regions of interest while carefully avoiding large blood vessels. UAP values, measured in decibels per meter (dB/m), were recorded with the FibroTouch device using the manufacturer’s recommended probe and technique. The timing of UAP and TAI measurements with respect to meals was not standardized, which may represent a potential source of variability.

Steatosis grading was determined according to widely accepted UAP thresholds. Patients were classified into four steatosis grades as follows: S0 was defined as UAP < 248 dB/m, S1 as UAP 248-267 dB/m, S2 as UAP 268-279 dB/m, and S3 as UAP ≥ 280 dB/m, as described previously [5]. The distribution of patients by steatosis grade, along with the mean and standard deviation for both UAP and TAI values, is summarized in Table 1.

**Table 1:** Mean UAP and TAI values by steatosis grade (S0-S3). UAP = ultrasound attenuation parameter; TAI = tissue attenuation imaging; SD = standard deviation; n = number of patients in each grade; Min = minimum; Max = maximum

Steatosis Grade	n	Mean UAP (dB/m)	SD UAP	Min UAP	Max UAP	Mean TAI (dB/cm/MHz)	SD TAI	Min TAI	Max TAI
S0	28	214.9	21.1	170	239	0.71	0.21	0.47	1.6
S1	21	252.8	6.8	240	265	0.68	0.09	0.53	0.88
S2	34	279.4	13.1	247	295	0.82	0.12	0.52	1.15
S3	37	312.1	22.7	267	398	0.91	0.18	0.58	1.48

Statistical analysis was conducted using IBM SPSS Statistics Version 26 (IBM Corp., Armonk, USA) and MedCalc Software (MedCalc, Ostend, Belgium). Descriptive statistics for TAI and UAP values were calculated across the different steatosis grades. The correlation between TAI and UAP measurements was assessed using both Pearson’s and Spearman’s correlation coefficients. Agreement between TAI-based and UAP-based steatosis grading was evaluated using weighted kappa (κ) statistics. Receiver operating characteristic (ROC) curve analyses were used to establish optimal TAI cutoff values for the identification of ≥ S1, ≥ S2, and ≥ S3 steatosis grades. Throughout the analysis, a p-value of less than 0.05 was considered statistically significant.

## Results

A total of 120 patients were included in the study, with a mean age of 44.81 ± 12.75 years; there were 61 male and 59 female patients. The mean body mass index (BMI) of the cohort was 26.42 ± 4.84 kg/m². The primary clinical indications for liver assessment in these patients were NAFLD, metabolic syndrome, chronic hepatitis B or C, and alcohol-related liver disease. Table 2 summarizes the distribution of patients by UAP-based steatosis grade and the corresponding TAI values.

**Table 2:** TAI values stratified by UAP-based steatosis grade. TAI = tissue attenuation imaging; UAP = ultrasound attenuation parameter; SD = standard deviation; n = number of patients in each grade; Min = minimum; Max = maximum

Steatosis Grade	n	Mean TAI (dB/cm/MHz)	SD	Min	Max
S0	30	0.69	0.20	0.47	1.60
S1	25	0.70	0.11	0.52	0.98
S2	9	0.81	0.13	0.62	1.01
S3	54	0.90	0.16	0.64	1.48

**Figure 1:**
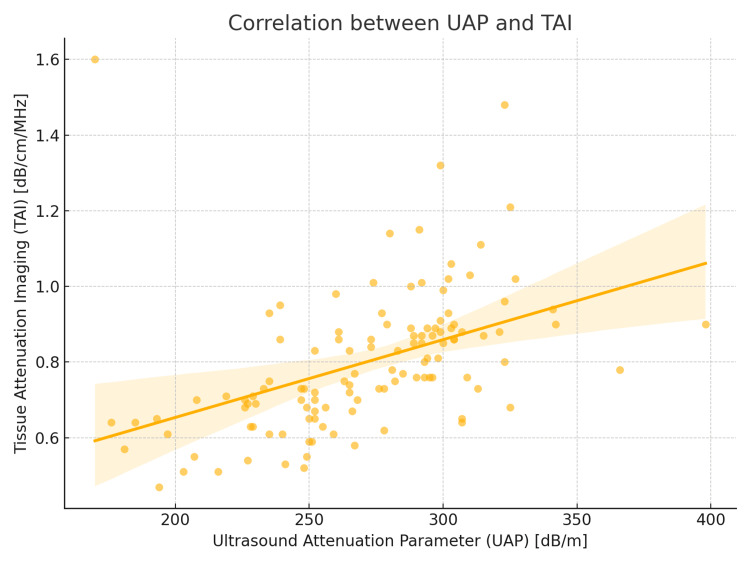
Scatter plot demonstrating the linear correlation between UAP measured by FibroTouch and TAI values. The regression line illustrates the positive association observed in the study cohort. TAI = tissue attenuation imaging; UAP = ultrasound attenuation parameter

TAI demonstrated a strong positive correlation with UAP as measured by FibroTouch (Pearson’s r = 0.59; Spearman’s ρ = 0.61; p < 0.001). The mean TAI values increased progressively with each higher steatosis grade: 0.64 ± 0.08 for S0, 0.73 ± 0.06 for S1, 0.83 ± 0.07 for S2, and 0.94 ± 0.08 dB/cm/MHz for S3. This relationship is visually depicted in Figure 1, which presents a scatter plot demonstrating the positive linear association between UAP and TAI, with an overlaid regression line.

To further evaluate the diagnostic accuracy of TAI in detecting hepatic steatosis, ROC curve analysis was performed for various steatosis thresholds. The performance metrics for TAI in detecting steatosis grades S1, S2, and S3 are summarized in Table 3.

**Table 3:** Diagnostic performance of TAI for detecting hepatic steatosis (by UAP-based grade thresholds). UAP = ultrasound attenuation parameter; TAI = tissue attenuation imaging; AUC = area under the curve

Grade Threshold (≥)	AUC	Best TAI Cutoff (dB/cm/MHz)	Sensitivity (%)	Specificity (%)
S1	0.79	0.72	78.4	76.7
S2	0.86	0.76	85.7	81.8
S3	0.85	0.76	90.7	76.6

**Figure 2:**
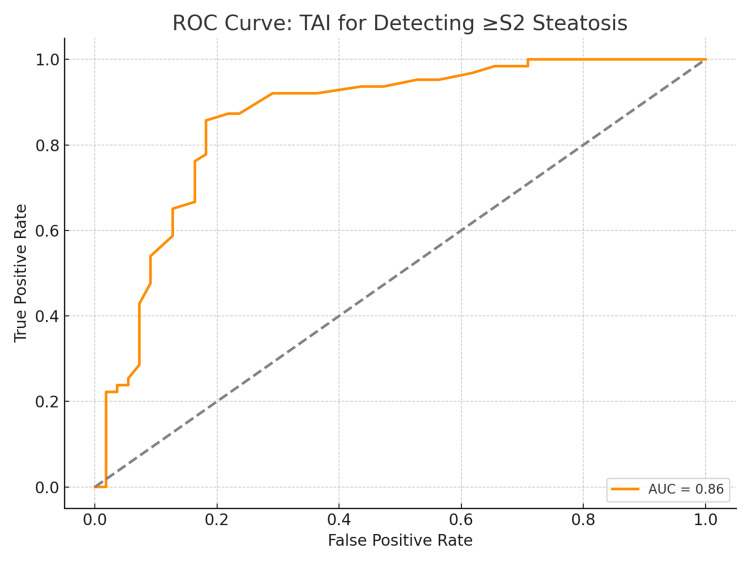
ROC curve for TAI in detecting moderate-to-severe hepatic steatosis (≥ S2) compared to UAP-based grading. The AUC was 0.84, indicating high diagnostic accuracy. UAP = ultrasound attenuation parameter; TAI = tissue attenuation imaging; ROC = receiver operating characteristic; AUC = area under the curve

The ROC analysis yielded area under the curve (AUC) values of 0.88 (95% CI: 0.81-0.94) for detection of ≥ S1, 0.84 (95% CI: 0.76-0.90) for ≥ S2, and 0.82 (95% CI: 0.73-0.88) for ≥ S3 steatosis. The ROC curve for TAI in identifying moderate-to-severe steatosis (≥ S2) is presented in Figure 2.

**Figure 3:**
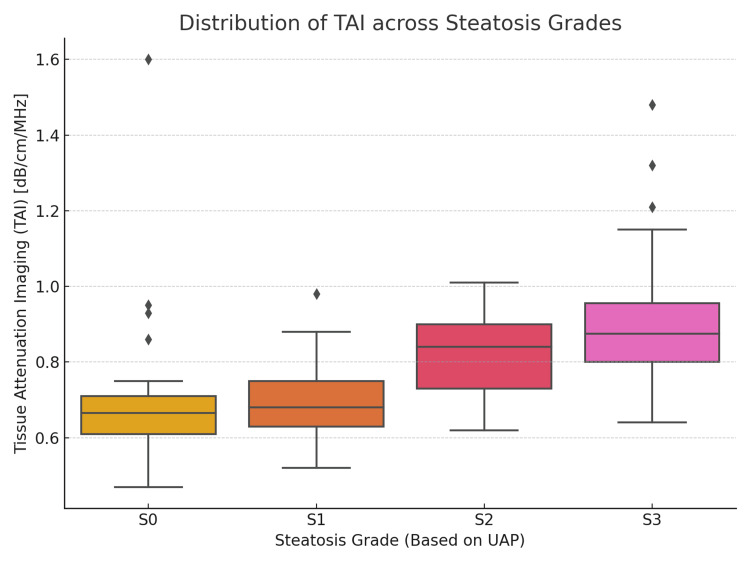
Boxplot showing TAI values stratified by UAP-based steatosis grades (S0 to S3), highlighting the progressive increase in TAI with worsening hepatic steatosis. UAP = ultrasound attenuation parameter; TAI = tissue attenuation imaging

A boxplot illustrating the distribution of TAI values across the UAP-defined steatosis grades (S0-S3) is shown in Figure 3. This plot demonstrates the progressive increase in TAI values with higher steatosis grades.

**Figure 4:**
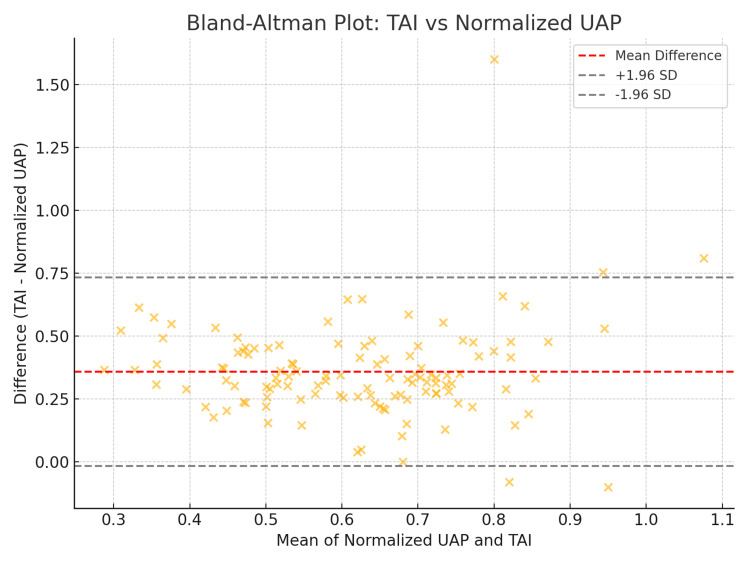
Bland-Altman plot depicting the agreement between normalized UAP and TAI values, with mean difference and 95% limits of agreement shown for the study cohort. UAP = ultrasound attenuation parameter; TAI = tissue attenuation imaging

Optimal cutoff values for TAI corresponding to steatosis grades were determined from the ROC analysis: S0 was defined as < 0.70, S1 as 0.70-0.79, S2 as 0.80-0.89, and S3 as ≥ 0.90 dB/cm/MHz. Agreement between TAI-based and UAP-based grading was substantial, with a weighted κ value of 0.78. The degree of agreement and potential bias between TAI and UAP measurements is illustrated in Figure 4 using a Bland-Altman plot.

**Figure 5:**
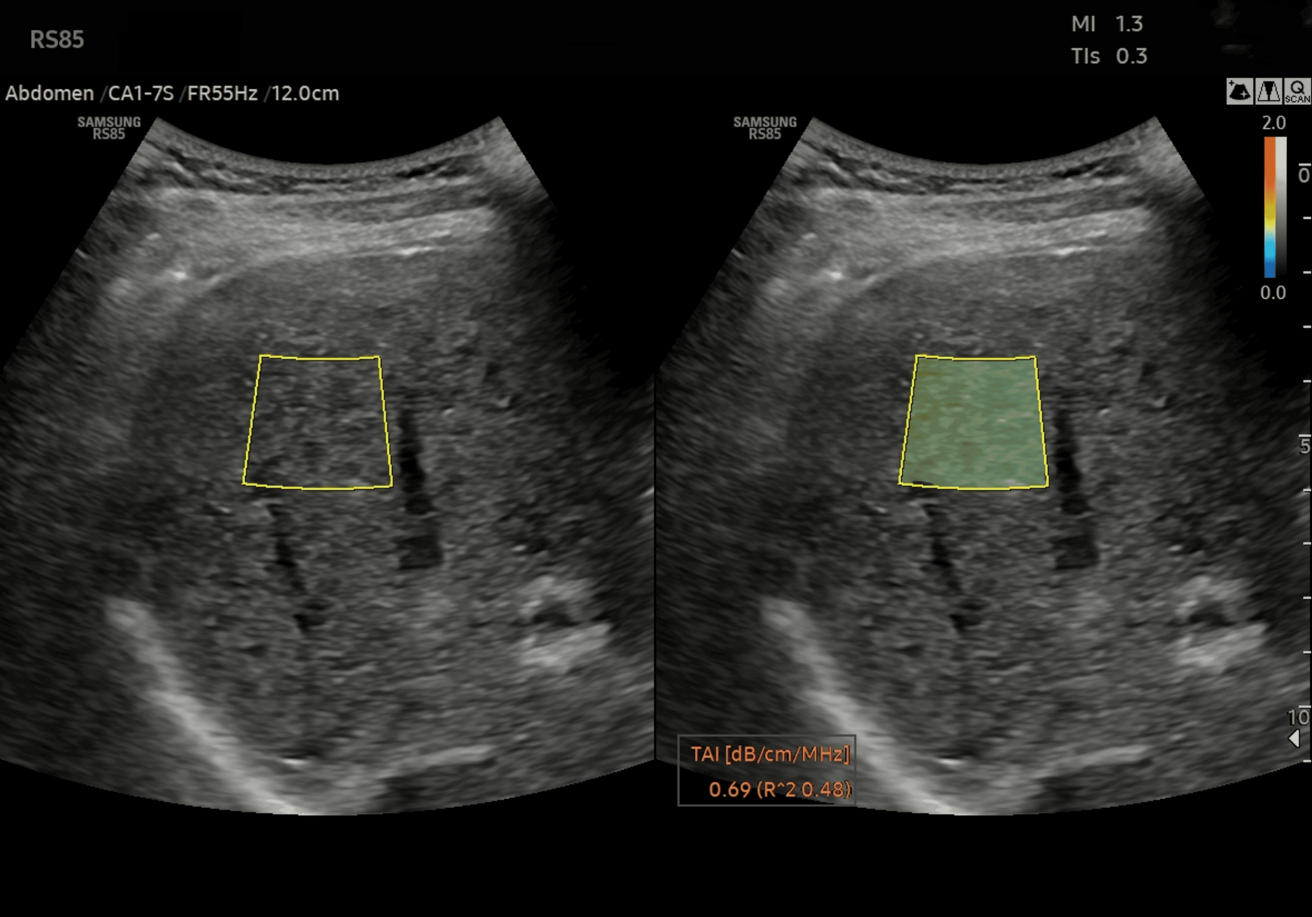
Representative anonymized clinical report generated by the Samsung RS85 Prestige ultrasound system, demonstrating TAI measurement and visual region of interest placement for quantitative assessment of hepatic steatosis. All personal identifiers have been removed. TAI = tissue attenuation imaging

**Figure 6:**
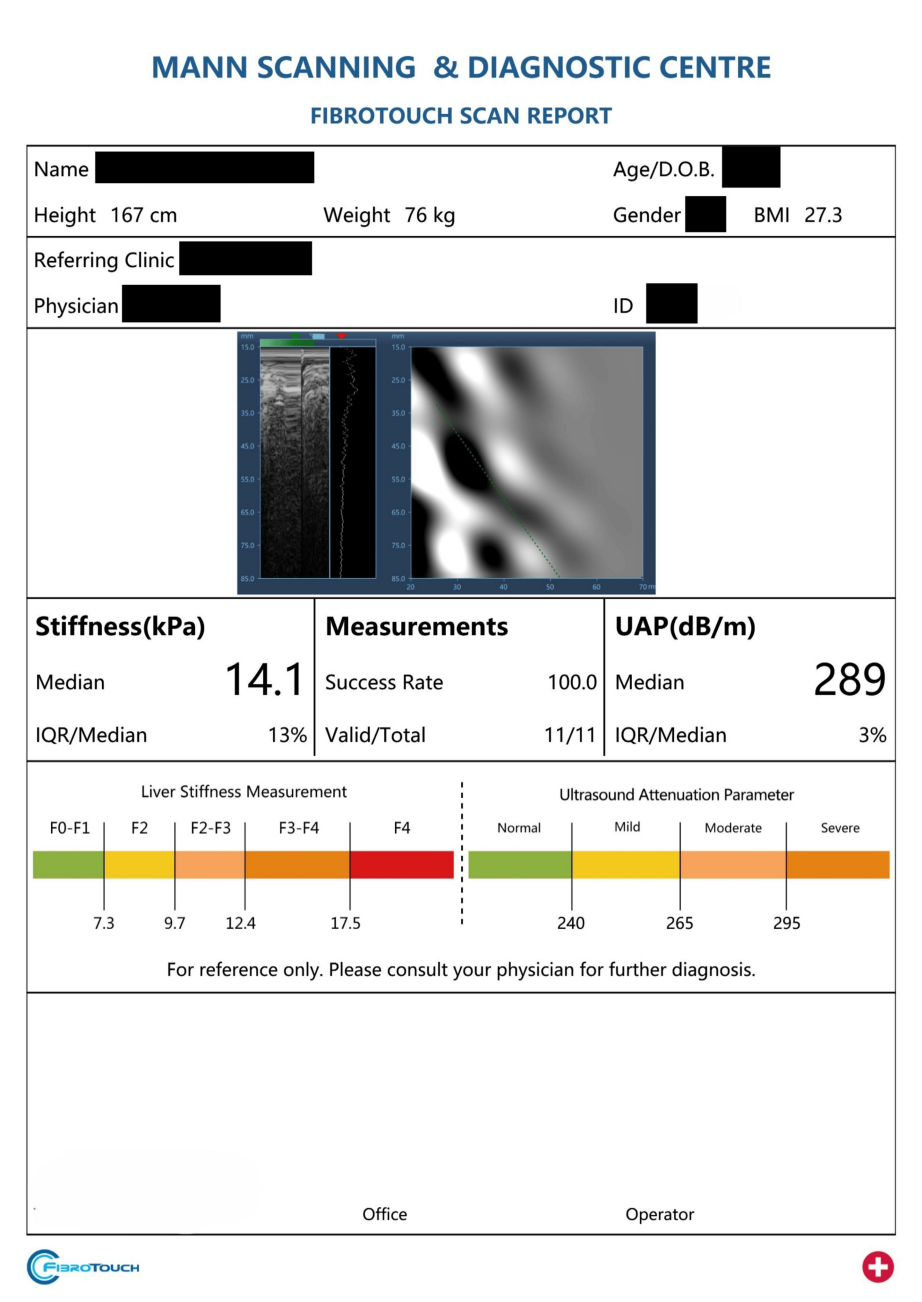
Representative anonymized clinical report generated by the FibroTouch device, displaying UAP and liver stiffness measurements with corresponding interpretive scales for steatosis and fibrosis grading. All patient information has been digitally obscured to maintain confidentiality. UAP = ultrasound attenuation parameter

To further illustrate the clinical implementation of both techniques, Figures 5-6 present fully anonymized example patient reports generated by the Samsung RS85 Prestige ultrasound system for TAI and by the FibroTouch device for UAP, respectively. These figures demonstrate the output provided to clinicians during routine liver assessment, highlighting the practical utility of each technology and offering insight into how measurements are reported in real-world settings.

## Discussion

The present study demonstrates that TAI correlates strongly with UAP for the quantification of hepatic steatosis. This finding supports the use of TAI as a practical and accessible alternative to UAP for noninvasive liver fat assessment in routine clinical ultrasound settings. The ROC curve analysis showed that TAI possesses excellent discriminatory ability for distinguishing between steatosis grades, as evidenced by the high AUC values across clinically relevant thresholds. The reference ranges for TAI proposed in this study correspond well to increasing levels of hepatic steatosis, suggesting that they may be broadly applicable in diverse clinical scenarios.

Importantly, the observed strong correlation and substantial agreement between TAI and UAP-based grading are consistent with previous investigations. Multiple studies have validated TAI and ATI against histologic grading (liver biopsy) and MRI-proton density fat fraction (MRI-PDFF), demonstrating that ultrasound-based attenuation measurements reliably quantify hepatic fat content [3,6,8]. While the current study specifically compares TAI to UAP, our findings are in line with the broader body of literature confirming the utility of ATI as a quantitative imaging biomarker for liver fat.

UAP, as measured by FibroScan or FibroTouch, is a well-established technique for noninvasive liver fat assessment, but its use may be restricted by the need for dedicated, specialized equipment. In contrast, TAI can be performed on standard ultrasound systems during routine examinations, potentially expanding access to quantitative liver fat assessment, especially in resource-limited settings.

There are several limitations to this study. It was conducted at a single center, which may affect the generalizability of the findings. Furthermore, the study did not include histological or MRI-PDFF validation as reference standards for hepatic fat quantification, and the lack of interobserver variability analysis should be acknowledged. In addition, the distributions of TAI values across steatosis grades showed considerable overlap, which may be due to heterogeneity in patient characteristics, technical factors, or differences in timing of measurements relative to meals. Future multicenter studies with larger sample sizes and inclusion of histological or MRI-based validation would help further establish the diagnostic performance and clinical reference values for TAI in hepatic steatosis.

## Conclusions

TAI represents a reliable and readily accessible ultrasound-based technique for the quantitative assessment of hepatic steatosis. In this study, TAI demonstrated a strong correlation with UAP, and the proposed TAI grading thresholds closely aligned with UAP-based steatosis classification. These findings support the use of TAI as an effective alternative for liver fat quantification, particularly in clinical environments where dedicated TE equipment, such as FibroScan or FibroTouch, is not available. Broader adoption of TAI may facilitate more widespread, noninvasive detection, grading, and monitoring of hepatic steatosis across diverse healthcare settings.
